# Comparing a Robot Tutee to a Human Tutee in a Learning-By-Teaching Scenario with Children

**DOI:** 10.3389/frobt.2022.836462

**Published:** 2022-02-21

**Authors:** Sofia Serholt, Sara Ekström, Dennis Küster, Sara Ljungblad, Lena Pareto

**Affiliations:** ^1^ Department of Applied IT, Division of Learning, Communication and IT, University of Gothenburg, Gothenburg, Sweden; ^2^ Division of Media and Design, School of Business, Economics and IT, University West, Trollhättan, Sweden; ^3^ University of Bremen, Bremen, Germany; ^4^ Department of Computer Science and Engineering, Interaction Design, University of Gothenburg and Chalmers University of Technology, Gothenburg, Sweden; ^5^ Department of Education, Communication and Learning, University of Gothenburg, Gothenburg, Sweden

**Keywords:** social robot, learning-by-teaching, in-the-wild, robot tutee, comparative study, children, robot versus human, child-robot interaction

## Abstract

Social robots are increasingly being studied in educational roles, including as tutees in learning-by-teaching applications. To explore the benefits and drawbacks of using robots in this way, it is important to study how robot tutees compare to traditional learning-by-teaching situations. In this paper, we report the results of a within-subjects field experiment that compared a robot tutee to a human tutee in a Swedish primary school. Sixth-grade students participated in the study as tutors in a collaborative mathematics game where they were responsible for teaching a robot tutee as well as a third-grade student in two separate sessions. Their teacher was present to provide support and guidance for both sessions. Participants’ perceptions of the interactions were then gathered through a set of quantitative instruments measuring their enjoyment and willingness to interact with the tutees again, communication and collaboration with the tutees, their understanding of the task, sense of autonomy as tutors, and perceived learning gains for tutor and tutee. The results showed that the two scenarios were comparable with respect to enjoyment and willingness to play again, as well as perceptions of learning gains. However, significant differences were found for communication and collaboration, which participants considered easier with a human tutee. They also felt significantly less autonomous in their roles as tutors with the robot tutee as measured by their stated need for their teacher’s help. Participants further appeared to perceive the activity as somewhat clearer and working better when playing with the human tutee. These findings suggest that children can enjoy engaging in peer tutoring with a robot tutee. However, the interactive capabilities of robots will need to improve quite substantially before they can potentially engage in autonomous and unsupervised interactions with children.

## Introduction

As robots are entering our social sphere (Gunkel, 2019), the question of how we should best (not) employ this new technology in everyday life has kindled many hopes and concerns (Bryson, 2010; Sharkey and Sharkey, 2010; Sharkey, 2016; Danaher, 2019; Sharkey et al., 2019; Nyholm and Smids, 2020). In recent years, research on child–robot interaction (CRI) and educational robots has begun to explore the possibility of using robots as tools for *learning-by-teaching* (Jamet et al., 2018). The idea to use physical robots for this purpose was first proposed by Tanaka and Kimura (2009) as a potential solution to ethical concerns surrounding the use of robots as teaching and childcare machines. Such robots have been referred to as teachable robots (Walker et al., 2016; Verhoeven et al., 2019), care-receiving robots (Tanaka and Matsuzoe, 2012; Nurul Husna et al., 2020), novice robots (Belpaeme et al., 2018), or robot tutees (Lindberg et al., 2017; Pareto, 2017; Chandra et al., 2020). The learning-by-teaching approach essentially reverses more traditional concepts. Early research on pedagogical virtual agents (Johnson et al., 2000; Johnson and Lester, 2016), in particular earlier studies of teachable agents (cf. Biswas et al., 2005), have paved the way for research on robot tutees. In conceptualizing these robots as in need of care (Tanaka and Kimura, 2009), the premise is that children will feel compelled to teach the robot. Hence, rather than acting as, say, a tutor directly teaching the child (cf. Kennedy et al., 2016b; Serholt and Barendregt, 2016; Jones and Castellano, 2018), a robot tutee is designed to feature as a curious and less knowledgeable other in need of the child’s teaching or instruction. Hence, inspired by the learning-by-teaching paradigm and the protégé effect^1^, the aim of such research is to evoke children’s drive to teach others, effectively fostering their own learning in the process.

Previous research on the effectiveness of robot tutees for child learning has delivered mixed results, and a variety of robot and research designs make it difficult to compare findings across projects. Some studies suggest that children’s learning can benefit from engaging with robot tutees. For instance, in a Japanese study, Tanaka and Matsuzoe (2012) found significant gains in English verb-learning for 3–5-year-old children following an experimental lesson in cases where children were also instructed to teach each word to a robot (as opposed to simply moving on to the next word). In a literature study on robot tutees, Jamet et al. (2018) argued for the relevance and efficiency of using robots as tutees for children of different ages as well as for children with special educational needs; however, no detailed information is provided on the literature review search protocol, making it difficult to assess the validity of these conclusions. In fact, research on robot tutees still seems to be relatively rare. In a meta-review of the literature on social robots in education, Belpaeme et al. (2018) found that only 9% of the 101 studies reviewed had explored the use of robots as tutees or peers.

One way to evaluate the effect of robots on learning and interaction is to experimentally compare them to other media or even humans. For instance, previous studies have compared robots acting as tutors to text-based learning systems and virtual agents (Pereira et al., 2008; Bainbridge et al., 2011; Leyzberg et al., 2012; Rosenthal-von der Pütten et al., 2016). Robots acting as tutees have been compared to virtual agents (Walker et al., 2016; Lindberg et al., 2017). However, only a few works have directly compared educational robots and humans (Belpaeme et al., 2018). Some of these have compared robots acting as tutors, instructors or interlocutors to humans (cf. Park et al., 2011; Serholt et al., 2014; Kennedy et al., 2016a; Kory Westlund et al., 2017). Another study compared a robot tutee to a human teacher, as well as to a tablet-only condition (Zhexenova et al., 2020). Chandra et al. (2015) compared a robot facilitating a learning-by-teaching scenario between two children to a human facilitator. The findings of these studies are presented in Section 2.3.

To our knowledge, no studies have attempted to compare robot tutees to human tutees. A possible explanation for this could be that it is methodologically difficult to recruit confederates that could serve as believable tutees while maintaining the necessary level of control over such an experiment. When robots have been directly compared to humans (see above), the human confederates have always been adults and these would probably not pass as convincing tutees. For the case of robot tutees that are designed for learning-by-teaching in a school setting, we propose that the proper baseline for comparison should be that of another child. In particular, we consider that the child tutee should be younger than the tutor, possibly instilling the presumption that they are not yet at the same level of proficiency as the child tutor concerning the subject or skill in question.

### Research Aim

Against this background, we set out to investigate how a learning-by-teaching situation with a robot tutee differs from an equivalent situation with a younger child. As this is the first such study, we adopt an explorative field-based experimental approach without a priori hypotheses. However, the following overarching research question and sub-questions guide this study:

RQ: How do children’s subjective experiences of tutoring a robot tutee compare to that of tutoring a younger child within the context of an educational game? In particular, what effects can be found pertaining to the child tutors’ perceptions regarding:

- 1) enjoyment and willingness to interact with the tutee again, 2) communication and collaboration with the tutee, 3) their understanding of the task, 4) their autonomy as tutors, and 5) learning gains for tutor and tutee, respectively?

The sub-questions are motivated as follows: 1) we consider enjoyment and willingness to engage with robots or similar educational technology to be prerequisites for their sustained use in future classrooms. Indeed, most CRI applications are motivated by their potential to increase children’s motivation and engagement by means of enjoyment. 2) It is also necessary for the communication and collaboration between tutor and tutee to work smoothly in order to avoid unmanageable technical issues and breakdowns in interaction. 3) We also consider the tutors’ understanding of the task and their role as tutor, and to what extent children intuitively understand what is expected of them in these scenarios. 4) Further, as many educational robots for children and adolescents are developed for the purpose of interacting with children autonomously and without too much external active involvement and assistance, the aspect of autonomy is included. Specifically, autonomy as tutors denotes to what extent the tutors perceive that they are able to handle their role as tutor without the help from teachers or researchers. 5) Finally, we explore the tutors’ assessments of subject learning gains—both their own, as well as the tutee’s. Here, their perceptions of tutee learning can also be understood as a reflection of how successful they find the overall interaction and their own tutoring.

## Background

In the following, we present literature on the concept of learning-by-teaching and its mechanisms. Then, previous research on robots in tutee roles is presented. Finally, we focus on previous studies that have experimentally compared robots to humans.

### Learning-By-Teaching

Originally developed by German professor Jean-Pol Martin, learning-by-teaching differs from similar peer-supported learning approaches in that students are directly responsible for both their own learning and the teaching of another (Aslan, 2015). This requires preparation (teaching oneself), selection of appropriate methods and materials for teaching, developing strategies that can motivate, engage and realize understanding in the tutee, as well as lesson design (Aslan, 2015).

Some research suggests that the learning benefits of learning-by-teaching lie in the preparation phase, i.e., if the tutor knows that (s)he is expected to engage in the direct teaching of novel material to a tutee without any teaching aids, they seem to engage with the teaching material more deeply, and hence, learn more (Koh et al., 2018; Kobayashi, 2019). However, while Koh et al. (2018) found that it was the retrieval process taking place during the preparing-to-teach phase itself which influenced learning outcomes for tutors, a study by Kobayashi (2021) found that the face-to-face interaction between tutor and tutee influenced learning gains meanwhile the preparation phase did not. Research further suggests that when it comes to difficult learning content, oral explaining seems to facilitate a tutor’s comprehension compared to if they compose written explanations (Jacob et al., 2020). Interestingly, with respect to written explanations, these seem to facilitate learning better if they are designed for self-explaining rather than as explanations for a fictitious tutee (Lachner et al., 2021).

Another interesting facet of learning-by-teaching is the demonstration of learning performed by tutees and its influence on tutor learning. For instance, Okita and Schwartz (2013) showed that tutors learned better when they observed their tutees performing examinations in biology following the tutor’s teaching. The same effect could be seen in a second experiment where the tutee was replaced by a teachable agent; i.e., those tutors who observed their agent compete against another agent exhibited higher learning gains compared to tutors who competed against their agent themselves (Okita and Schwartz, 2013). Similar outcomes were seen in a study that took place in a virtual game environment, i.e., tutors who observed their tutees perform an examination following their teaching learned better themselves (Okita et al., 2013).

While studies such as these are typically carried out with adults (e.g., university students), Hoogerheide et al. (2019) conducted a study with Dutch primary school students between 11 and 13 years old. The authors compared three conditions, namely: a restudy, a summarizing, and a video condition. In the video condition, the children were instructed to record a teaching video for a fictitious tutee after studying a text on biology over a weekend. In the summarizing condition, children instead produced written summaries of the text. In the restudy condition, children were simply asked to repeatedly read through the text. The study found that children who only restudied the text performed the worst on a posttest across the three conditions. Yet, contrary to the authors’ expectations, the difference between written summaries and teaching videos was not straightforward. There seemed to be some effect, i.e., the video condition was significantly more effective than the restudy condition, whereas the summarizing condition was not; however, there was no significant difference when comparing the summarizing and video condition (Hoogerheide et al., 2019).

### Robots as Tutees

Previous research on robot tutees has tended to focus on educational subject areas where the tutor is presumed to already hold a certain level of knowledge, minimizing the necessity for tutors to prepare and design appropriate lessons themselves. Notwithstanding, it is not clear to what extent mechanisms of learning-by-teaching transfer from human peer tutoring to robots. While there seem to be some general effects on learning, this also depends on what the robot tutee is compared to. For instance, in the earlier example of a study by Tanaka and Matsuzoe (2012), significant learning effects were indeed observed for children who taught words to a robot compared to those who did not. Yet, the control condition in this case may not be comparable since the children simply moved on in the task and did not practice the words as those interacting with the robot invariably did. In the study by Chandra et al. (2020), significant improvement in children’s handwriting was only observed in a condition where the robot tutee was improving between sessions, compared to a condition where it did not improve. Lindberg et al. (2017) conducted a within-subjects comparison between a robot tutee and a teachable agent, and mainly found individual differences between children. In a similar vein, children’s rapport towards a robot tutee has been shown to fluctuate both between children and over time (Tian et al., 2020), and, e.g., children’s pre-existing comfort levels with robots seem to influence their acceptance of a robot tutee’s social behavior (Lubold et al., 2019). In one of our previous studies, we found that children employ a range of strategies to repair interactions with a robot tutee behaving in socially inappropriate ways, ranging from trying to understand and adapt to the robot, to establishing a social distance to it (Serholt et al., 2020). Further, Lemaignan et al. (2016) observed promising effects pertaining to engagement and learning gains when using a handwriting tutee robot for children’s long-term occupational therapy; however, they also emphasized the need for further studies on the ethical implications surrounding the child–robot relationship that could develop, i.e., the potential psychological implications for children who commit to helping a robot. Chen et al. (2020) compared a robot tutee to either a robot tutor or a robot peer (i.e., an adaptive combination of the tutee/tutor role) and found that children’s learning gains were superior to the tutee condition in both the peer and tutor conditions. Walker et al. (2016) found that middle school students significantly improved their geometry knowledge following a lesson with a teachable robot within a navigation task on an interactive tabletop surface, but this was also the case in conditions where the robot’s face was displayed on a stationary monitor and the physical robot was replaced with a projected circle, as well as for students who instead carried out the task with a virtual agent and a laptop.

It is worth noting that applications of robot tutees do not necessarily require machine learning approaches wherein the robot is *truly* learning; yet, it is likely important that the robot *appears* to be learning on a level convincing for the child teaching it, or, perhaps, that the child is willing to engage in make-believe with the robot through a temporary suspension of disbelief. Early in our project, when children in the second and fourth grade (7–10 year-olds; *N* = 67) interacted with a prototype version of the robot used in the current study, 61% indicated that they believed that they were teaching the robot, whereas 27% considered the teaching reciprocal, and 3% believed that the robot was teaching them (Pareto et al., 2019). Further, early pilot studies carried out within a project using a co-writing robot tutee found that none of the participating 7–8 year-old children (*N* = 21) questioned whether the robot tutee was writing on its own (Hood et al., 2015), which may suggest that they believed it was improving in response to their instruction. At the same time, it does not seem to matter much whether the robot tutee indeed improves during the interaction for children to believe that it does. For instance, in the case of a co-writing robot tutee, Chandra et al. (2020) found that 7–9-year-old children believed that the robot was progressing equally well in its writing abilities over the course of four sessions regardless of its actual improvement. Here, the authors compared different versions of the robot in two studies: 1) non-learning versus learning between sessions (*N* = 25), and 2) non-learning, personalized learning and continuous learning (*N* = 37). In terms of the children’s perceptions of their own self-efficacy as tutors, no significant differences were revealed between any of these conditions.

Regardless of observed effects, it is important to note the importance of an active adult presence (e.g., teachers, experimenters or therapists) during these interactions (Lemaignan et al., 2016), and the possible confounding effects these actors may have on overall outcomes. Due to technical constraints in current robots, the general consensus is that children cannot be expected to manage interactions with robots on their own, although there are exceptions (cf. Davison et al., 2020). There are also ethical constraints that make it questionable whether leaving children alone with robots is a good idea in relation to their physical and emotional safety (Serholt et al., 2017; Serholt, 2018). Some applications are simply not designed to work without some level of continuous guidance by an expert.

### Robots Compared to Humans

As mentioned previously, only a few studies have specifically explored how social robots compare to humans. To our knowledge, the first such study was conducted about 30 years ago by Draper and Clayton (1992) wherein they explored how a Heathkit Hero 1 robot compared to a set of other conditions, including a human teacher, a tape recorder, a stationary robot, and a control condition. The robot had been decorated with facial features in foam rubber, and been given a human recorded voice instead of the standard voice. Children ages 3–5 took part in the study, which centered on a lesson about birds and a posttest in the form of 20-min interviews. With respect to children’s learning gains, the human teacher clearly outperformed the other conditions. Unsurprisingly, learning gains in the robot conditions were significantly better than the control condition. In terms of children’s lesson attention (as measured by gaze towards either the instructor or visual aids), they were the most attentive towards the teacher (91%), followed by the animated robot (88%), the stationary robot (78%), and finally, the tape recorder (60%). However, the difference between attention levels towards the teacher and animated robot was not statistically significant (Draper and Clayton, 1992). Thus, it seems that the robot was able to capture the children’s attention, but perhaps not teach them like the human teacher could.

While not many studies were done immediately after 1992, comparative studies of robots and humans have received an upswing in the past decade. For instance, Park et al. (2011) conducted a between-subjects comparative experiment with university students (*N* = 126) aged 19–33 in Seoul, Korea, where the students attended a 10-min history lecture about the Renaissance era followed by a knowledge test. Independent variables included either a robot or a human instructor, as well as different forms of random feedback on the participants’ posttest performances (positive, neutral or negative). The results of the study revealed that the students were generally more accepting towards receiving feedback from the human instructor. In terms of attraction to the instructor, the students were significantly less attracted to the robot instructor when it provided negative feedback; however, the same effect did not apply to the human instructor (Park et al., 2011).

With respect to children, studies comparing robots to human instructors/tutors have been conducted with children ranging from the younger ages of 2–5 (Moriguchi et al., 2010; Moriguchi et al., 2011; Kory Westlund et al., 2017), and 6–9 (Chandra et al., 2015; Kennedy et al., 2016a; Zhexenova et al., 2020), to 11–15-year olds (Serholt et al., 2014).

The studies with younger children focused on the learning of novel words (Moriguchi et al., 2011; Kory Westlund et al., 2017) or to what extent a robot versus a human could influence children’s behavior (Moriguchi et al., 2010). In the latter case, perseverative behaviors in children as influenced by either a robot’s or a human’s demonstration of how to sort a deck of cards (on video) were investigated. Specifically, the human/robot would sort the cards according to either color or shape. Then, an experimenter asked the children to sort the cards opposite to what they had observed on the video (i.e., if they had watched color sorting, they were asked to sort the cards according to shape, and vice versa). Interestingly, the results revealed a significant influence of the human demonstration on children, leading them to produce significantly more mistakes compared to the robot condition as well as a baseline condition. Similarly, but in terms of children’s word-learning, Moriguchi et al. (2011) found that children learned new words significantly better from a human compared to a robot even though the children only watched videos of either a human or a robot labeling different objects. In contrast, Kory Westlund et al. (2017) found no significant differences between a robot and a human with respect to children’s learning of novel words. Here, it might be the case that the embodiment and presence of the different robots played a role. While the studies by Draper and Clayton (1992) and Moriguchi et al. (2011) used quite mechanical-looking robots (i.e., Hero 1 and Robovie), Kory Westlund et al. (2017) used a version of the MIT-developed Tega robot. The Tega robot has a zoomorphic and fluffy appearance with a high-pitched voice, and probably resembles a stuffed toy more than the stereotypical image of a robot.

In relation to children between six and 9 years old, Kennedy et al. (2016a) compared a robot tutor to a human tutor in the context of a lesson on prime numbers. A between-subject design was employed with children (*N* = 22) aged around 8 years old. The results showed that both experimental groups had improved significantly on a post-test, and while there were no significant differences between the robot and the human, differences in effect size were noteworthy. Thus, the authors concluded that the experiment partially confirmed their hypothesis that children learned better with a human tutor. Moreover, Chandra et al. (2015) conducted a between-subjects experiment in a Portuguese school with 6–8-year-old students (*N* = 40) in which they compared a robot to a human facilitator in a learning-by-teaching situation. However, unlike the current study with a robot tutee compared against a child tutee, their scenario looked at a facilitator role. Specifically, pairs of children were randomly assigned to either a teacher or a learner role in an interactive writing session where the child in the teacher role was tasked to provide feedback to the learner following his or her writing. Each session was facilitated by either a robot or a human who would ask the teacher-child to provide feedback. The results indicated that the child in the teacher role took more responsibility for the learner-child when a robot facilitated the session as demonstrated by more elaborate feedback. Significant learning gains were seen for both groups, and there were no significant differences between them. Further, in a study using a robot tutee, Zhexenova et al. (2020) found that children aged seven to nine from a school in Kazakhstan significantly improved their learning of Latin script letters when teaching them to a robot. Yet, their learning was only slightly higher than children who only used a tablet, and lower than a group taught by a human teacher; none of these findings were statistically significant. With respect to subjective experiences, the robot received the highest likeability rating, the teacher received the highest effectiveness rating, but no statistical differences were found for interest and easiness (Zhexenova et al., 2020).

Looking at older children and adolescents, Serholt et al. (2014) carried out a between-subjects comparative experiment of a robot tutor and a human tutor with 11–15-year-old students (*N* = 27) in a Swedish school. The children were tasked to follow step-by-step instructions in constructing a small and simple house using LEGO-bricks from either tutor. The study found that the children performed equally well in terms of task success in both conditions. They tried to request help from the human tutor, whereas this never happened with the robot. While signs of engagement were significantly higher for the human condition, children in the robot condition were significantly keener on performing well. In terms of changes in pre- and post-test attitudes toward the robot’s scores, children in both conditions became significantly more positive about situations and interactions with robots, and the social influence of robots. As concerns differences between conditions, the children in the robot condition became more positive towards conducting their schoolwork with a robot, and they also assigned a higher level of conceptual meaning to the word ‘robot’ following the experiment (Serholt et al., 2014).

The aforementioned studies all share the fact that a robot was compared to a human adult. The current study thus addresses a clear gap in the empirical literature by comparing a robot tutee to children as tutees.

## Materials and Methods

In this small-scale field experiment, we explored how 12 to 13-year-old students (sixth-graders) perceive a game-based peer-tutoring activity depending on if the tutee is either a social robot (Pepper from Softbank Robotics) or a 9 to 10-year-old child (a third-grader). In this paper, we present the results of the tutors’ subjective responses to a set of measures administered after the experiment.

### The Student Tutor and Robot Tutee System

**FIGURE 1:**
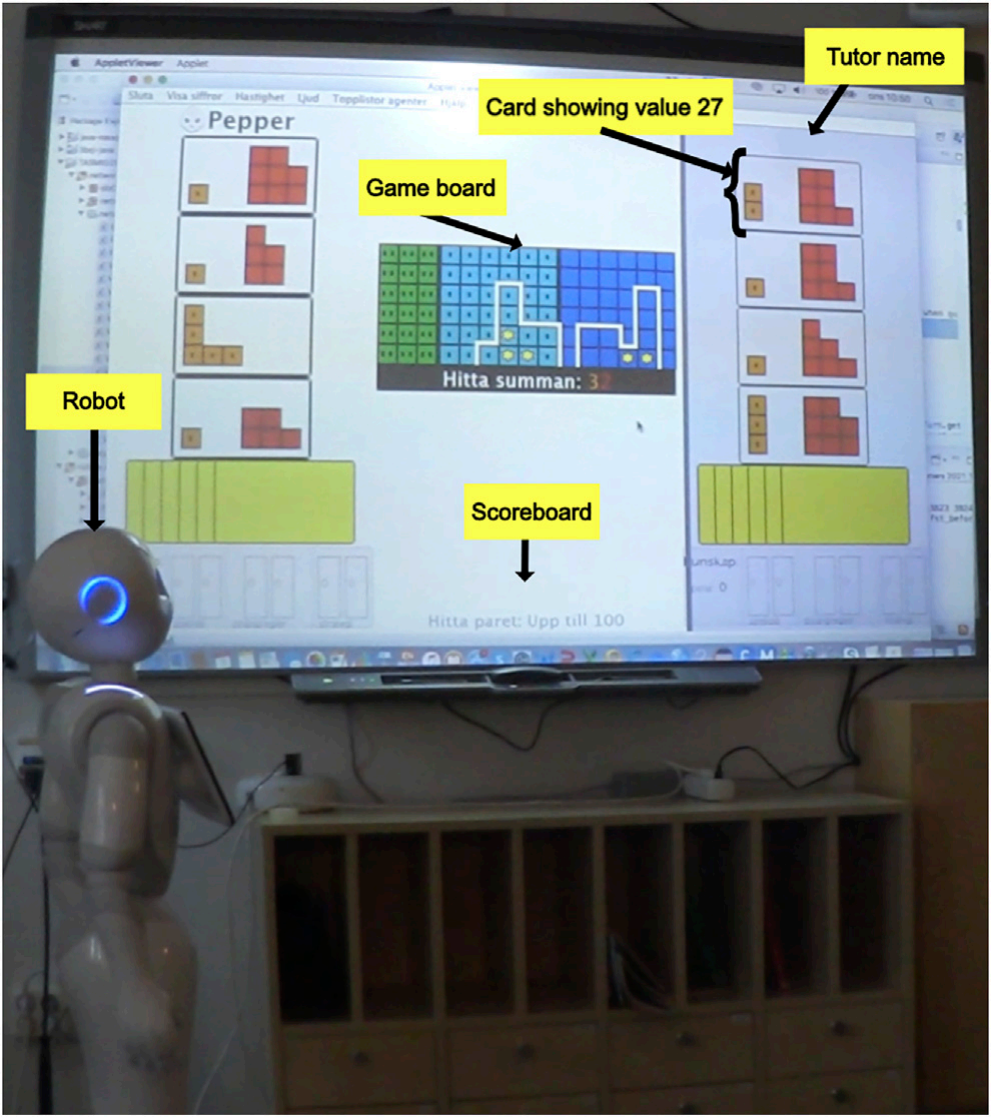
The START System showing the card hands and visible cards for the tutee and tutor on the left- and right-hand side, respectively. The tutor name has been redacted to preserve the participant’s anonymity.

The experiment was carried out as an evaluation study of the robot system developed within the Student Tutor and Robot Tutee (START) project (Pareto et al., 2019; Serholt et al., 2020). The aim of the START project has been to explore the potentials of robot tutees in education by augmenting an existing game in mathematics with a robot tutee (Pareto et al., 2019). Specifically, the project sought to 1) develop an effective robotic learning companion, 2) leverage learning of the educational content by combining game-based and inquiry-based learning, and 3) model ideal learner behavior such as scientific inquiry reasoning for the tutor to be inspired by and to imitate (Pareto and Barendregt, 2016). The selected game has previously been shown to be highly effective for computational, conceptual, and strategic thinking in mathematics learning (Pareto et al., 2011; Pareto, 2014). The original system was based on the same learning-by-teaching approach, but rather than a robot tutee, it utilized a virtual text-based agent without any social behavior. The START System thus consists of a wall-mounted interactive whiteboard displaying the educational game, and a social robot tutee (Pepper from Softbank Robotics) placed towards the left side of the screen (see Figure 1). In a previous study using an earlier version of the system, we explored interaction trouble and repair (Serholt et al., 2020).

The game is called the Graphical Arithmetic Game, and it is a 2-player card game in which mathematical values are graphically represented by colored blocks on an interactive whiteboard or display (Pareto, 2014). The players take turns playing their respective cards, with the goal to reach a designated value displayed on a joint game board. The game comprises a set of mini-games based on identical gaming principles. In this particular experiment, we used a mini-game called “Find the Pair up to 100”. The aim of the game is to choose the two cards that equal the sum of whatever value is displayed on the screen (max = 100). Each round consists of the players playing two cards, one from each player. There are always four cards available to choose from in each player’s hand, which means that there are 16 possible combinations for each round. The blocks differ in colors depending on if they represent values in tens (orange) or ones (red). The players discuss and agree on an appropriate match of cards for each round. Once a card is selected from the active player’s side of the screen, it transfers over to the game board through an animation visualizing the number of blocks. It is then the other player’s turn to select a matching card. Once a card is played, it cannot be withdrawn, so the players need to discuss which two cards to choose before they play, in order to play well. Hence, each game round gives rise to strategic discussions regarding methods to find the pair, e.g., by excluding all cards greater than the sum, but also approximate as well as exact mental calculations to judge a proposed pair. There is a span of difficulty levels within the task of mental integer addition depending on the two integers involved in the sum (Buijsman and Pantsar, 2020). For each correct pair, a star is added to the scoreboard. If the answer is incorrect, a dialog box appears signaling this. The game is completed when the two players have played ten rounds.

The robot’s social behavioral repertoire has been developed through a co-design process with children (Barendregt et al., 2020), and with a particular focus on designing the robot to behave in a way believable for a tutee. In this final version of the system, the robot’s behavior includes gestures, gazing, a text-to-speech engine and automatic speech recognition in Swedish for verbal communication. The robot is connected to the game through a local wireless network such that the robot’s behavior is contingent on the tutor’s actions in the game (selection of cards) and interaction with the tutee (responses to questions). Throughout the game, the robot asks inquisitive questions about the game mechanics and the current arithmetic problem. Depending on how well the tutor manages to answer the robot’s question, upcoming questions can increase or decrease in difficulty. The robot can also restate a previously unanswered question, provide positive feedback on the tutor’s teaching abilities, and express enjoyment. At the beginning of the game, the robot is at a novice stage and does not play its own cards yet. Instead, it asks basic questions or makes random “guesses” as to what card it should try and play and that the tutor then selects. After approximately 10 min of gameplay, the robot begins selecting cards on its own after asking the tutor for approval.

### Research Design

The experiment was conducted in an empty classroom at a primary school in Sweden. In order to compare the two educational scenarios, the experiment followed a within-subjects design, in which the order of both conditions, robot tutee (RT) and child tutee (CT), were counterbalanced for half of the participants. The second condition was conducted as a separate session, 2 days after the first session.

### Participants

Twenty students at the school participated in the study; ten sixth-graders as tutors and ten third-graders as tutees. As explained previously, this study focuses on the subjective experiences of the sixth-graders (*N* = 10; 5 girls; 12–13-year-olds) in their roles as tutors. The sixth-graders had previous experience both playing the Graphical Arithmetic Game (albeit some of the easier mini-games) and interacting with the robot during the iterative development and participatory design process of the START project (Pareto et al., 2019; Barendregt et al., 2020). They had also taken part in a previous study with the mathematics game and an earlier version of the RT in groups with their classmates (Serholt et al., 2020). Consequently, the sixth-graders were familiar with the concept that they served as tutors in this scenario.

### Procedure

Prior to the study, children’s legal guardians provided written informed consent, whereas the children provided written assent. Each of the participating children was allocated a 35-min time slot per experimental condition. First, the participant was invited to the experimental classroom. In the CT condition, the sixth- and third-grader were invited to the experimental classroom together where they were introduced to each other; no additional familiarization phase was held for the tutor–tutee pairs and we did not investigate if any of the pairs knew each other beforehand. In the experimental classroom, participants were given a brief introduction to the experiment, including their roles as tutors responsible for guiding and teaching the game to the CT and RT, respectively. Participants were then asked to confirm their assent to be video-recorded, whereby the game was started. As mentioned previously, each game consisted of ten rounds. In cases where there was time left after the first game, the participants were given the opportunity to play again.

During both conditions, the sixth-graders’ mathematics teacher was present to provide guidance and support when needed, and at her own discretion. In addition, two researchers handled the technical equipment and data collection. Thereafter, debriefing sessions were held during which the sixth-graders responded to questionnaires measuring their perceptions of the interactions.

### Measures

We administered three types of quantitative instruments for gauging participants’ subjective perceptions of the interactions: 1) a custom 18-item evaluation questionnaire with responses given on a 5-point ordinal scale ranging from *strongly disagree—strongly agree*; 2) a 4-item visual analogue scale (VAS); and 3) a 5-item *Again and Again* (A&A) table. The VAS and A&A table were both adapted from the Fun Toolkit, which is a set of validated instruments developed by Read (2008) for evaluating technology with children, whereas the questionnaire was developed by the research team. All measures were designed or adapted for the purpose of addressing our research question and for evaluating the START system against the target aims of the overall research project. The questionnaire was administered directly after each condition, whereas the VAS and A&A were administered upon completion of both conditions.

In addition, logs detailing time duration, number of rounds, and number of points were extracted from the game. Video recordings of the interaction sessions will be analyzed and presented in a separate publication.

#### Evaluation Questionnaire

The 18-item questionnaire was developed jointly by the research team. It includes nine pairs of items (see Table 1) measuring enjoyment, how understandable and clear the task felt, how well the tutor and tutee collaborated, how well they communicated or understood each other, as well as a subjective rating of the quality of the tutee’s questions. It also measured to what extent they felt in need of the teacher’s presence, as well as how much help they felt they needed from the teacher. Finally, it measured perceptions of learning: both the tutor’s own learning, as well as the tutee’s learning. For all items, we used 5-point Likert scales^2^, with the most negative response anchors on the left (i.e., *strongly disagree, disagree, neither, agree,* and *strongly agree*). To increase reliability and avoid swaying the participants to answer in a socially desirable way, inverted (negatively keyed) items were included for all items (cf. Paulhus, 1991). This resulted in 9 positively and 9 negatively worded items for all constructs. The ordering of the items was randomized to avoid presenting positively or negatively framed items in any particular order. The questionnaires for the two conditions were identical apart from the references to the tutee (RT = Pepper; CT = younger student).

**TABLE 1:** Items on the two evaluation questionnaires (RT = Pepper; CT = younger student).

Construct	Item	Inverse
1. Enjoyment	It was fun to play with Pepper/younger student	It was boring to play with Pepper/younger student*
2. Clarity of task	It was clear what I was supposed to do	It was unclear what I was supposed to do*
3. Collaboration	Pepper/younger student and I collaborated well	It was hard to collaborate with Pepper/younger student*
4. Communication	Me and Pepper/younger student understood each other well	Me and Pepper/younger student **did not** understand each other*
5. Quality of questions	I thought Pepper/younger student asked good questions	I thought Pepper/younger student should have asked better questions*
6. Need for teacher presence	It was unnecessary that the teacher was there	It was good that the teacher was there*
7. Need for teacher help	I **did not** need help from the teacher to play with Pepper/younger student	I needed help from the teacher to play with Pepper/younger student*
8. Tutee learning^a^	I think I taught Pepper/younger student mathematics	I **do not** think Pepper/younger student learned any mathematics from me*
9. Tutor learning	I got better at mathematics by playing with Pepper/younger student	My knowledge in mathematics **did not** improve by playing with Pepper/younger student*

a While it may appear as though the items for Construct 8 are asymmetrical in the use of “taught” versus “learned”, it should be noted that the Swedish translation for “taught” is the same as “learn”, i.e., tutee learning is implicit in both items.

Prior to the study, the questionnaire was piloted with a child in the sixth grade through a think-aloud approach, which suggested that the questions were clear and easy to understand. However, some of the inverted items containing the formulation “not/do not” were misread when read quickly. To address this, such negations were formatted in bold font.

#### Visual Analogue Scale

The visual analogue scale (VAS) used for this study comprised four items to gather a continuous measure of enjoyment, how well the activity worked, perceptions of own learning, and how much the teacher needed to help. Our implementation of this VAS was loosely based on the Fun Sorter from the Fun Toolkit (Read, 2008). Each item contained pictures illustrating the two conditions both above and below the analogue rating scale. The participants’ task was to draw a line from each picture to the scale line in the center. The lines were designed as semantic differential scales, containing antonyms at each end (*very boring–very fun; very bad–very good; very little–very much*). Items are presented in Table 2.

**TABLE 2:** VAS items.

Construct	Item	Semantic differential scale
1. Enjoyment	What did you think of the activity?	Very boring—very fun
2. Task experience	How did you think the activity worked?	Very bad—very good
7. Need for teacher help	How much did the teacher need to help?	Very little—very much
9. Tutor learning	How much mathematics did you learn?	Very little—very much

#### Again and Again Table

The Again and Again (A&A) table used for this study was again adapted from the Fun Toolkit (Read, 2008). An A&A table asks a straightforward question at the top of a table (i.e., *“Would you like to do the following again?”*). In the present study, the participants responded by providing a checkmark in the appropriate box (*Yes, maybe,* or *no*) next to a set of scenarios illustrated through pictures and a written description below each picture. Each of the five items (except for the first) consisted of scenarios they had encountered previously, i.e.: 1) playing the math game on their own, 2) playing the math game with the RT, 3) playing the math game with the CT, 4) playing the math game with the RT and classmates, and 5) hanging out with the RT without playing the math game. The A&A table is available as supplementary material.

### Data Processing and Analysis

Data were processed and analyzed using IBM SPSS 28. First, ratings of negatively worded items were recoded for both the questionnaire and the VAS, such that higher values reflect positive judgments. We likewise recoded responses for the items concerning help from the teacher to simplify our analysis, i.e., low scores thus indicate that the participant needed/received a lot of help.

For the questionnaire, we then calculated the mean of the original and inversed item pairs to obtain a composite score for each of the nine constructs. As recommended by Eisinga et al. (2013), we report Spearman-Brown reliability estimates, since each of our scales comprised two congeneric items. Despite the small number of items, the reliability of most constructs was acceptable or higher for most of our constructs (enjoyment *ρ = 0.843;* collaboration *ρ = 0.902;* communication *ρ = 0.883;* quality of questions *ρ = 0.797;* need for teacher presence *ρ = 0.803;* teacher help *ρ = 0.551;* tutee learning *ρ = 0.661;* tutor learning *ρ = 0.824*). The estimated reliability of one construct was less than acceptable^3^ (clarity of task *ρ = 0.183*). We nevertheless retained this construct for conceptual reasons.

For the VAS, we measured participants’ responses in millimeters from the center of the line, with negative values denoting a negative response. We then calculated the percentage of each response based on the full length of the scale line from the center. If the participant had drawn a line extending past the full length of the scale line in either direction, this was recorded as ± 100%, which occurred in 5/80 responses.

Given the small sample size, we opted to limit our analysis to non-parametric statistical testing. As this was a within-subjects design, we focus mainly on individual differences and related samples testing using Wilcoxon signed-rank tests. We also calculated effect sizes to show the strengths of effects.

## Results

The game logs showed that the participants spent between 12 min 33 s and 34 min 21 s actively playing the game with the tutees. The average game time appeared to be slightly higher in the RT condition (*M* = 26 min 29 s; *SD* = 06 min 8 s) than in the CT condition (*M* = 24 min 20 s; *SD* = 4 min 51 s). However, this difference was not statistically significant (Z = −0.764, *p* = 0.445, *r* = −0.17). Similarly, the frequency of correct answers were higher on average in the CT condition (*M* = 95.5; *SD* = 4.97) compared to the RT condition (*M* = 91.17; *SD* = 15.11). However, these differences were not statistically significant either (Z = −0.843, *p* = 0.399; *r* = −0.19).

### Enjoyment and Interaction Willingness

With respect to enjoyment of the interaction, children generally indicated that they were very positive towards both the child and robot tutee on the questionnaire administered immediately following each condition: *M* = 4.8, *SD* = 0.42 for the CT, and *M* = 4.7, *SD* = 0.42 for the RT. Thus, enjoyment reported by the children appeared to reflect a ceiling effect, suggesting that children very much enjoyed playing the game with either type of tutee. On the VAS administered at the end of the study, enjoyment ratings for the CT and RT were likewise positive, although there did not appear to be a ceiling effect for this measure: *M* = 72.01, *SD* = 21.07 for the CT and *M* = 72.44, *SD* = 23.29 for the RT (i.e., on a scale from -100 to 100). Wilcoxon signed-rank tests did not indicate any significant differences across conditions on either the questionnaire (*Z* = -0.816, *p* = 0.414, *r* = -0.18) or the VAS (*Z* = -0.070, *p* = 0.944, *r* = -0.02).

**FIGURE 2:**
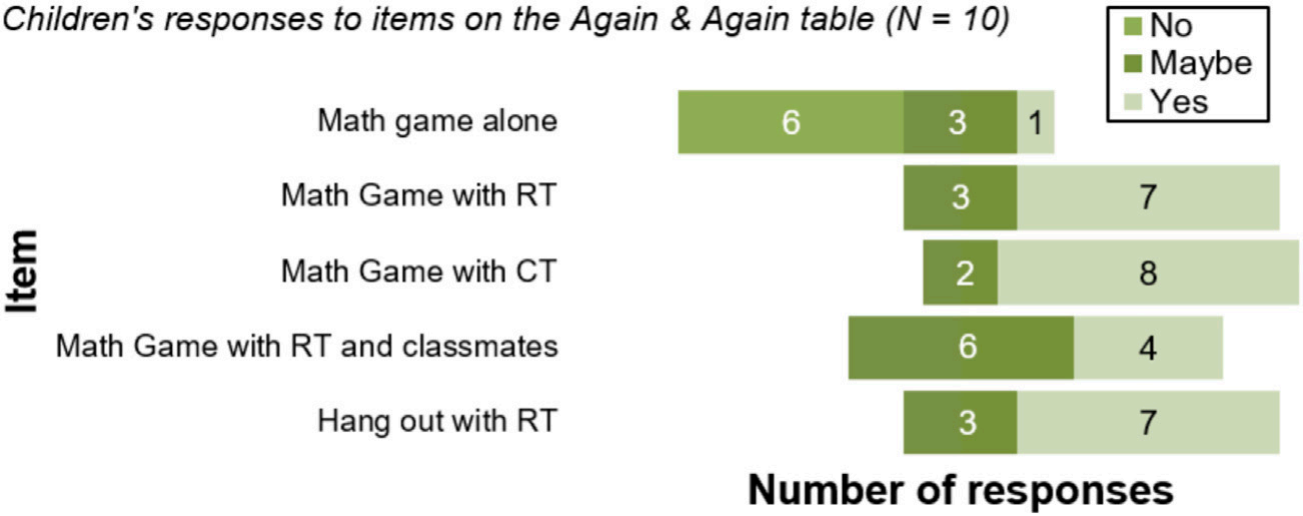
Diverging bar chart showing frequencies of children’s responses to the set of questions on the A&A table.

In the A&A table, all children responded either “yes” or “maybe” to wanting to play the math game again with the RT and the CT (see Figure 2). Similar responses were seen for their willingness to hang out with the RT without playing the game. However, they were slightly less certain towards playing the math game with the RT and their classmates as they had done in our previous study (Serholt et al., 2020). A majority were disinterested in playing the math game alone, suggesting a strong motivational contribution of the interaction with a tutee during the game. There were no significant differences between their willingness to play the game again with the CT and the RT (Z = −0.577, *p* = 0.564, *r* = −0.13), or to play the game again with the CT and to hang out with the RT (Z = −0.577, *p* = 0.564, *r* = −0.13).

### Communication and Collaboration

**FIGURE 3:**
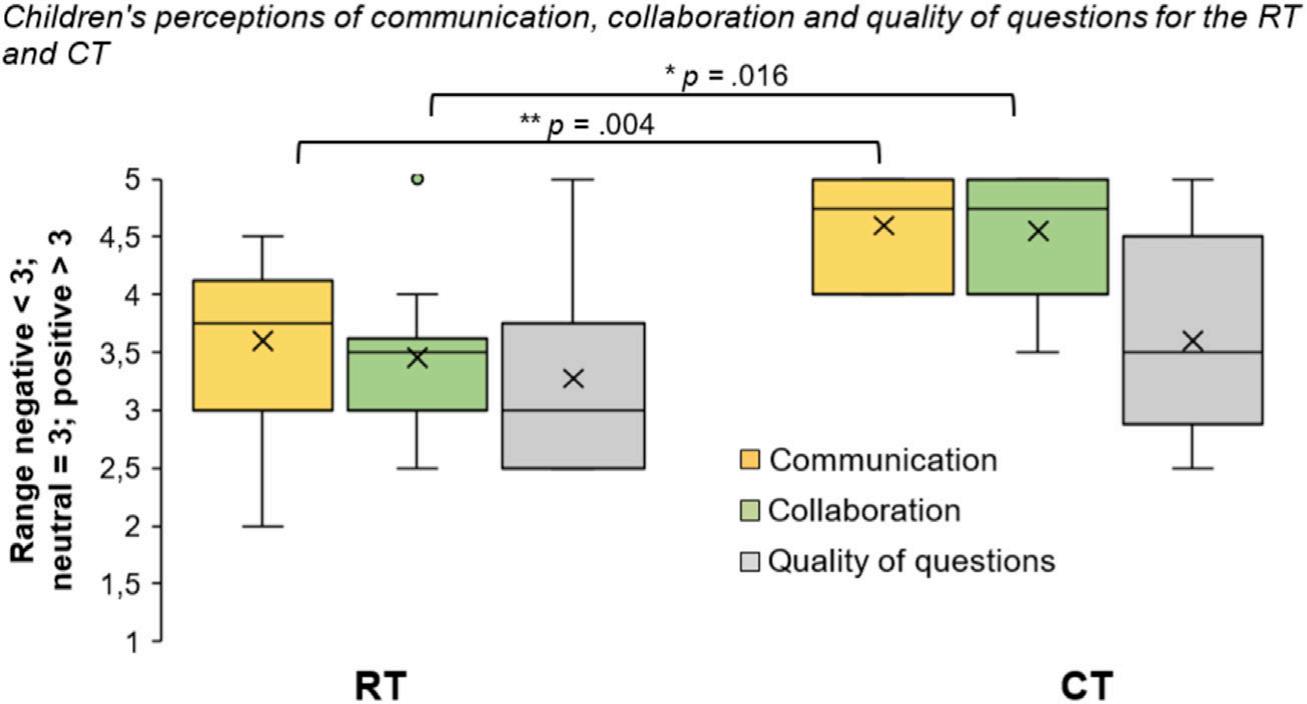
Box plots showing children’s perceptions of ease of communication, collaboration and quality of questions for the RT and CT (x = means; lines = medians; whiskers = ranges). *p < 0.05. **p < 0.01.

As can be seen from Figure 3, children’s perceptions of the tutees in terms of ease of communication and collaboration were generally more positive towards the CT. The differences were significant with large effect sizes for questions pertaining to both collaboration (*Z* = −2.399, *p* = 0.016, *r* = −0.54) and communication (*Z* = −2.844, *p* = 0.004, *r* = −0.64). However, children’s perceptions of the quality of the tutees’ questions did not differ significantly between the RT and the CT (*Z* = −0.780, *p* = 0.435, *r* = −0.18), although it is worth noting that there was a missing value for one participant on this construct in the RT condition.

### Task Clarity and Experience

**FIGURE 4:**
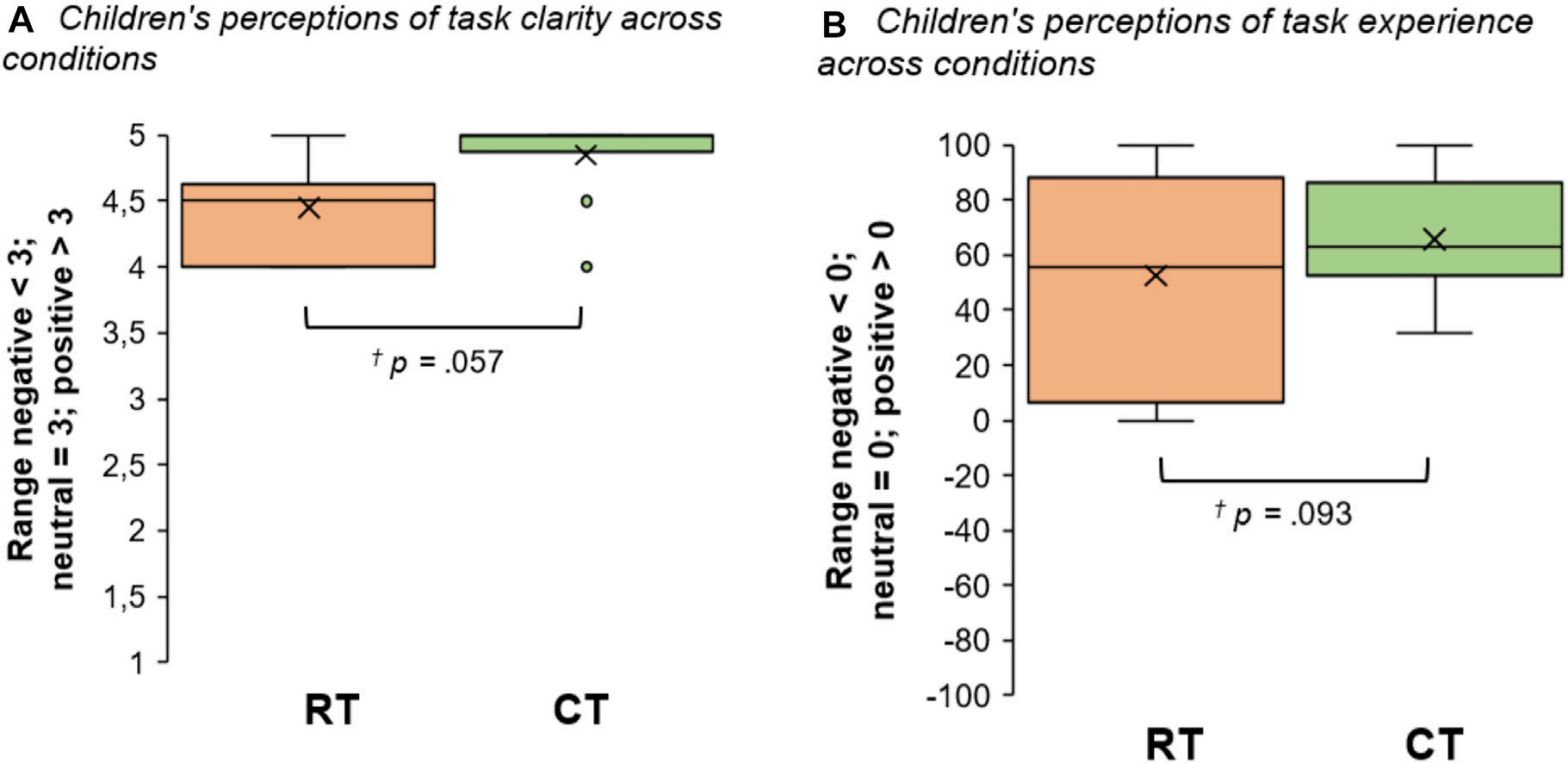
Box plots showing children’s responses to **(A)** questionnaire items on task clarity, and **(B)** VAS items on how well the task worked, both across conditions (x = means; lines = medians; whiskers = ranges).

On the questionnaires and the VAS, children were asked to rate how clear they felt that the overall task was (task clarity), and their experience of how well it worked (task experience). In terms of clarity, they appeared to be slightly more positive towards the interaction with the CT (*M* = 4.85, *SD* = 0.34) compared to the RT (*M* = 4.45, *SD* = 0.37) (see Figure 4A); however, statistically, these findings were marginally significant with a moderate effect size (*Z* = −1.903, *p* = 0.057, *r* = −0.43). In terms of how well the task worked, on average, they appeared to perceive the interaction with the CT somewhat more positively than the RT (*M*_CT_ = 65.91, *SD* = 20.86 versus *M*_RT_ = 52.49, *SD* = 38.43) (see Figure 4B). Again, these differences were marginally significant with a moderate effect size (*Z* = −1.680, *p* = 0.093, *r* = −0.38).

### Tutor Autonomy

**FIGURE 5:**
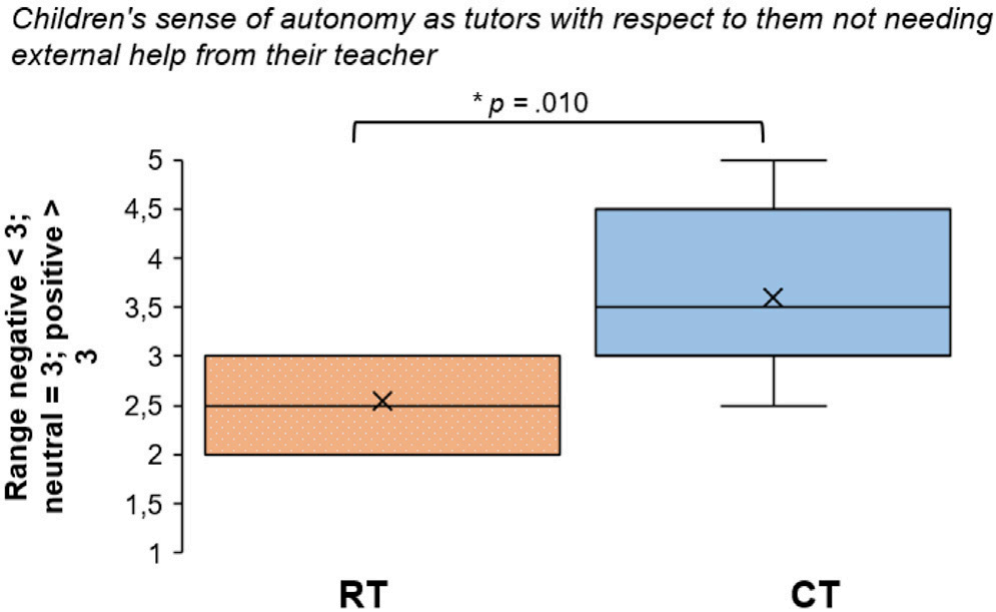
Box plots showing comparisons of composite scores for inverted items regarding need for teacher help, hence signifying the tutor’s sense of autonomy for the RT and the CT (x = means; lines = medians; whiskers = ranges).

As mentioned in Section 3.6, children’s perceptions of their autonomy as tutors were assessed based on their stated need for teacher presence and help. On the evaluation questionnaires, children indicated a low sense of autonomy related to the presence of the teacher (*M*_RT_ = 1.80, *SD* = 0.71; *M*_CT_ = 2.20, *SD* = 0.71), as well as external help (*M*_RT_ = 2.55, SD = 0.44; *M*_CT_ = 3.60, *SD* = 0.810). This suggests that they did not find it feasible to act as tutors and handle the sessions without teacher support, particularly not with the RT. There were no significant differences between conditions concerning the need for teacher presence, albeit a moderate effect size (*Z* = −1.358, *p* = 0.174, *r* = −0.30). However, the difference for teacher help was significant, such that the tutor fared better without help in the CT condition compared to the RT condition (*Z* = −2.573, *p* = 0.010, *r* = −0.58; see Figure 5). On the VAS, responses were more spread out and unevenly ranked (*M*_RT_ = 24.11, *SD* = 54.72; *M*_CT_ = 43.57, *SD* = 40.78); thus, a small effect size with no significant differences was found (*Z* = −1.172, *p* = 0.241, *r* = −0.26).

### Learning Gains

**FIGURE 6:**
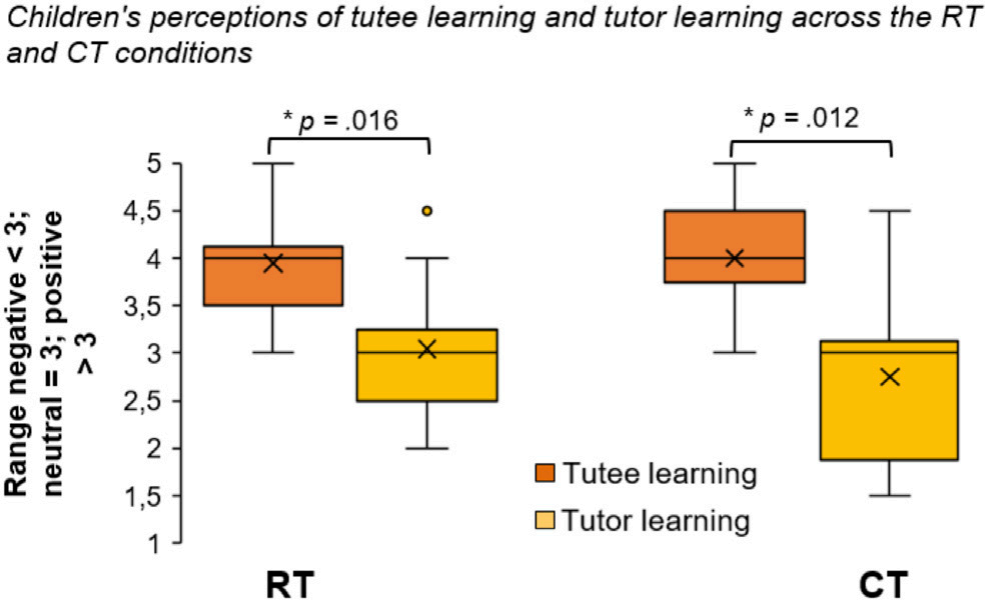
Box plot (from questionnaires) showing children’s perceptions of tutee and tutor learning gains in mathematics across conditions (x = means; lines = medians; whiskers = ranges).

Finally, perceived learning gains for the tutee as well as for the tutor were assessed through the questionnaires and the VAS. Here, children indicated that they believed the RT and CT learned almost equally well (*M*_RT_ = 3.95, *SD* = 0.55 compared to *M*_CT_ = 4.00, *SD* = 0.62; *Z* = -0.431, *p* = 0.666, *r* = −0.10). Concerning perceptions of the tutors’ own learning gains in mathematics, responses were lower for both the RT and CT compared to perceived tutee learning (*M*_RT_ = 3.05, *SD* = 0.72; *M*_CT_ = 2.75, *SD* = 0.92). These differences were significant with large effect sizes within both the RT (*Z* = −2.401; *p* = 0.016, *r* = −0.54) and the CT (*Z* = −2.501; *p* = 0.012, *r* = −0.56) conditions. On the VAS, many responses were on the negative end of the scale with respect to perceived learning gains of the tutor (*M*_RT_ = −0.56, *SD* = 57.13; *M*_CT_ = 1.33, *SD* = 63.38). In other words, while children generally believed that the tutees experienced some learning gains, they were much less inclined to consider that they themselves improved their mathematics learning in their roles as tutors (see Figure 6). With respect to perceptions of tutor learning gains across the two conditions, there were no significant differences between conditions on either the questionnaires (*Z* = −1.604, *p* = 0.109, *r* = −0.36) or the VAS (*Z* = −0.280, *p* = 0.779; *r* = −0.06), although there was a moderate effect size on the questionnaire suggesting that the tutor felt they learned more with the RT than with the CT.

## Discussion

This is the first study to systematically compare robots to human tutees and children in particular. Earlier studies have until now only compared robots to adults in teaching or facilitator roles, and typically only used one particular person, meaning that results are highly dependent on the personality of that individual, and, perhaps, his or her previous experience with children. By involving different children as tutees for comparison, we were able to circumvent this limitation and focus more on the learning-by-teaching situation itself. As demonstrated by a growing number of studies applying the learning-by-teaching approach (Koh et al., 2018; Kobayashi, 2021) being in the tutoring role can be a very effective learning instrument, so it is important to study the mechanisms beneficial for learning-by-teaching such as when it involves robot tutees.

Our results suggest high enjoyment with the activity regardless of the tutee condition. The teacher’s presence was highly valued in both cases; however, the need for teacher help was more pronounced in the robot condition. As discussed in previous studies, it is important to consider the presence of adults such as teachers or therapists during CRI (Lemaignan et al., 2016), and what influence they may have on the interaction and experience (cf. Serholt, 2018; Serholt et al., 2020). Had the teacher not been present during the interaction sessions (particularly with the robot tutee), this study would likely have yielded a different set of results. Indeed, video data obtained from the study which is currently being analyzed suggests that the teacher was highly involved in the interaction, also with some technical support from the researchers running the experiment. Such active involvement of adults provides some guarantee that the experiments could run smoothly, without which children’s subjective experiences of the sessions may have been less positive overall.

Further, the tutors did not consider that they themselves learned very much from the activity; yet they thought that both the child and the robot tutee learned some mathematics as a result of their teaching. This is interesting in relation to believability in the learning-by-teaching scenario, i.e., that the tutee appears to learn (Hood et al., 2015; Pareto et al., 2019). Even so, since beliefs that the tutee was learning in response to their teaching did not spill over to assessments of their own learning, this may suggest that these are two rather distinct and possibly unrelated mechanisms in learning-by-teaching. Another explanation could be that the game was simply a bit too easy for a sixth-grader in that it only focused on mental arithmetic. This is supported by the fact that the frequencies of correct answers were fairly high for most tutor–tutee pairs.

Furthermore, overall experiences pertaining to the task and how clear it felt differed somewhat in favor of the session with the younger child. We further observed significant differences between conditions in relation to perceptions of tutor–tutee collaboration and understanding, both in favor of the younger child. This can be expected, as verbal communication with fully autonomous robots is very difficult to achieve due to technical limitations in current speech recognition technology—particularly the speech recognition used in off-the-shelf robots (Belpaeme et al., 2018; Serholt et al., 2020). There will most likely always be limitations in how well AI can collaborate and socialize with people, due to computational limitations of understanding and relating to the world (Broussard, 2018). Furthermore, limited experiences of interacting with robots can affect the interaction as people are not sure what to expect (Thunberg and Ziemke, 2020). Since communication is a prerequisite for collaboration to work, it is not surprising that children find it easier to interact with another child. This supports and supplements previous research that has compared robots to human adults, suggesting that robots are found highly likeable by children, but may not measure up to a human in other regards (Serholt et al., 2014; Kennedy et al., 2016a; Zhexenova et al., 2020).

### Limitations and Future Work

This paper’s results are based on empirical data consisting of children’s subjective perceptions through quantitative questionnaires. We have not studied how the interactions unfolded, and are therefore unable to provide any contextual explanations for participants’ responses. This limits the conclusions that can be drawn from these results. In future work, we will conduct video analysis of the interaction sessions, which will be discussed in relation to these findings.

Another limitation of this study was its small sample size. This was due to the fact that this research coincided with the corona pandemic, which limited possibilities for conducting research in schools. Although we have accounted for this in our statistical analysis, we advise future work comparing robots to humans to recruit more participants.

Moreover, enjoyment reported by the participants appeared to reflect a ceiling effect in the questionnaire. However, there was no appearance of a ceiling effect on the enjoyment measure for the VAS. This suggests that continuous measures could be helpful for questionnaire items that tend to produce ceiling effects.

Finally, the Spearman-Brown correlation coefficients for one of the constructs on the evaluation questionnaire was quite low. While our small sample size limits the extent to which these reliability estimates can be meaningfully interpreted (cf. Bonett and Wright, 2000), it is possible that some children responded in a socially desirable way on positively keyed questions whereas they were more honest when a question was negatively framed (or vice versa). It could also be the case that some children simply misread some of the items and interpreted them as the opposite of what was intended, despite our use of bold font for negations. Notwithstanding these considerations, future work could aim to include scales with a larger number of items to increase robustness (Eisinga et al., 2013). This should, of course, be balanced against the risk of making the questionnaire too long and tiresome for children; e.g., one might then consider limiting the number of constructs.

## Conclusion

In this study, we compared a robot tutee to a human tutee in the context of a collaborative mathematics game with the rationale that humans can serve as baselines for comparison and, hence, evaluation of a robot’s feasibility in whatever context it may be designed for. Such studies are still quite few and far in between, and the reasons for this may partly be related to the fact that researchers tend to view robots as supplementary to human practices rather than as replacements. Even so, robots will invariably replace some aspects of the educational practice, and it is therefore important to explore how close a robot can come to more traditional approaches.

Overall, our study illustrated that learning-by-teaching is a new and challenging task, and sixth-graders still required and appreciated the guidance and support offered by their teacher. Furthermore, it is perhaps not surprising that the children in our study, overall, tended to perceive tutoring another child as easier compared to tutoring a robot. Nevertheless, it is remarkable how similar and positively both robot and child tutees were perceived in this study. For example, children attributed substantial learning gains in mathematics to the robot tutee—and these learning gains were perceived as exceeding their own learning gains, just as was the case for child tutees. Together, these findings suggest that the robot tutee could generally function in this role, albeit somewhat less well as another child. Indeed, we argue, it may not be necessary or even necessarily desirable for the robot tutee to match or outperform a child tutee in all of these respects. Instead, if future social robots become sufficiently adept for specific tutee roles, children may feel comfortable using them to practice their own teaching abilities.

Notwithstanding, it is important that any use of robots in education is subjected to a cost-benefit analysis and considered in light of current practices. If learning-by-teaching and peer tutoring is an effective method for learning, and can provide benefits for both tutor and tutee, such practices could be implemented more often in schools if they are not already even without robots—particularly if learning-by-teaching involving younger students is preferred by children. Of course, collaborations across school grades and curricula are not always possible to orchestrate. Yet, this should also be contrasted against the costs and practical difficulties in providing pedagogical staff for CRI-sessions with individual students. Given children’s low sense of autonomy in tutoring a robot themselves, along with the ethical implications of leaving children alone with robots, we anticipate that unsupervised and unguided CRI-sessions will continue to be unrealistic for the foreseeable future.

## Data Availability

The raw data supporting the conclusions of this article will be made available by the authors, without undue reservation.

## Footnotes

1 The protégé effect is a psychological phenomenon in which students make greater efforts to learn for the benefit of a tutee (or protégé) than they would for themselves (Chase et al., 2009; Lemaignan, et al., 2016).

2 While we considered using a smiley face ordinal response scale, as is common practice for studies with children (Hall et al., 2016), we opted to avoid this since there is a lack of research that has explored the appropriateness of using smiley face response scales for negatively worded items. For instance, one could argue that it might feel counterintuitive to respond to a statement such as “Playing with the robot was boring” with a happy face to indicate agreement. The fact that the participants were around 12 years old also suggests a level of maturity for comprehending conventional Likert-type ordinal scales.

3 Due to the small sample size, we estimated *ρ* based on the pooled ratings from both experimental conditions (CT, RT).
