# Supplementary material for: Comparing a Robot Tutee to a Human Tutee in a Learning-By-Teaching Scenario with Children
Source: Front Robot AI. 2022 Feb 21;9:836462. doi: 10.3389/frobt.2022.836462 (PMC8899022; doi:10.3389/frobt.2022.836462)
Supplement: Supplementary file 1 [file DataSheet1.pdf]

Supplementary Material

Would you like to do any of the following again?	**yes**	**maybe**	**no**
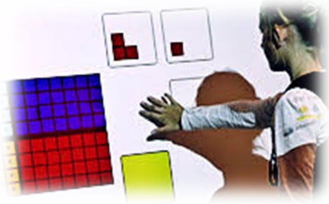 Play the math game by myself			
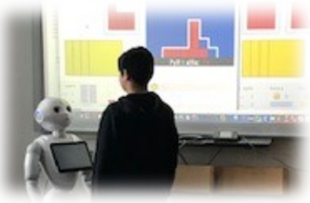 Play the math game with Pepper			
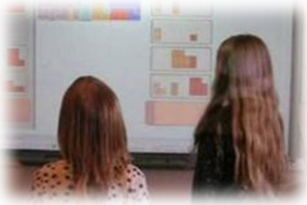 Play the math game with a younger student			

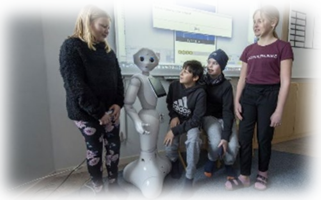 Play the math game with Pepper and classmates			
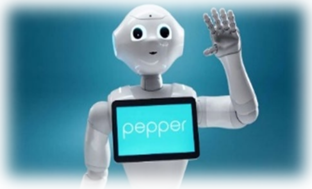 Hang out with Pepper without playing the math game			

Figure 1. Again & Again table administered to participants after the experiment.
